# Diagnostic Value of Serum IgG4 for IgG4-Related Disease: A PRISMA-compliant Systematic Review and Meta-analysis

**DOI:** 10.1097/MD.0000000000003785

**Published:** 2016-05-27

**Authors:** Mingju Hao, Min Liu, Gaowei Fan, Xin Yang, Jinming Li

**Affiliations:** From the National Center for Clinical Laboratories (MH, GF, XY, JL), Beijing Hospital; Graduate School (MH, GF, XY, JL), Peking Union Medical College, Chinese Academy of Medical Sciences, Beijing; Department of Clinical Laboratory (MH), Qianfo Mountain Hospital of Shandong University; and Department of Clinical Laboratory (ML), Jinan Dermatosis Prevention and Control Hospital, Jinan, People's Republic of China.

## Abstract

Many studies about serum IgG4 for the diagnosis of IgG4-related disease (IgG4-RD) have been reported. However, these studies had relatively small sample sizes and the diagnostic accuracy values varied much between them.

The aim of this study was to perform a meta-analysis to evaluate the diagnostic value of serum IgG4 for IgG4-RD.

We conducted a search of relevant articles using MEDLINE, EMBASE, Web of Science, SCOPUS, and Cochrane Library databases published before December 2015.

Studies those assessed the diagnostic accuracy of serum IgG4 for IgG4-RD and those provided the cut-off value for serum IgG4 were included.

Data were synthesized using the random-effect model. Statistical analysis was performed using STATA with the MIDAS module and Meta-DiSc 1.4 software.

A total of 9 case-control studies were analyzed, which included 1235 patients with IgG4-RD and 5696 overall controls. The pooled estimate, for a cut-off value ranged from 135 to 144 mg/dL, produced a sensitivity of 87.2% (95% CI, 85.2–89.0%) and a specificity of 82.6% (95% CI, 81.6–83.6%). The positive likelihood ratio (PLR), negative likelihood ratio (NLR), and diagnostic odds ratio (DOR) were 6.48 (95% CI, 3.98–10.57), 0.14 (95% CI, 0.09–0.21), and 45.15 (95% CI, 23.41–87.06), respectively. The area under the curve (AUC) of the summary receiver operating characteristic curve (SROC) was 0.94 (0.92–0.96). When a cut-off value of 2-fold the upper limit of normal was used (ranged from 270 to 280 mg/dL), the pooled sensitivity was 63% (95% CI, 60.0–66.0%), and the specificity was 94.8% (95% CI, 94.1–95.4%). The PLR, NLR, and DOR were 13.3 (95% CI, 7.39–24.0), 0.41 (95% CI, 0.29–0.58) and 33.42 (95% CI, 13.88–80.43), respectively. The AUC of the SROC was 0.92 (0.90–0.94).

Only a relatively small number of studies were included, and significant heterogeneity was observed in this meta-analysis.

Serum IgG4 is a modestly effective marker to diagnose IgG4-RD. Doubling the cut-off value for IgG4 could not improve the overall test characteristics. A high specificity inevitably accompanies with a significant sacrifice in sensitivity.

## INTRODUCTION

IgG4-related disease is an immune-mediated condition that can affect almost any organ.^1^ The characteristic features include a lymphoplasmacytic infiltrate composed of IgG4 positive plasma cells, storiform fibrosis, obliterative phlebitis, and mild-to-moderate eosinophilia.^1^ Most patients with IgG4-RD have elevated serum IgG4 concentrations and abundant accumulation of IgG4-positive plasma cells in affected tissues.^2^

Elevated serum levels of IgG4 provide a noninvasive method to diagnose IgG4-RD. The comprehensive diagnostic criteria for IgG4-RD contained 3 major criteria: (1) clinical characteristics of diffuse/localized swelling or masses in single or multiple organs; (2) serum IgG4 concentrations ≥135 mg/dL; (3) histopathologic characteristics of marked lymphocyte and plasmacyte infiltration and fibrosis, infiltration of IgG4-positive plasma cells: >40% of IgG4/IgG positive plasma cells and >10 IgG4 positive cells/high powered field of biopsy sample.^3,4^ Early reports associated IgG4-RD with an elevated serum IgG4 concentration demonstrated at least one-third of patients have a normal serum IgG4 concentration.^5–7^ These findings force us to question the actual significance of IgG4 in this condition.

So far, a wide range of diagnostic accuracy values of serum IgG4 for the diagnosis of IgG4-RD have been reported, and these studies have had relatively small sample sizes.^7–15^ Some previous meta-analysis studies focused on autoimmune pancreatitis (AIP)-the typical IgG4-RD.^16,17^ But these AIP patients were not clearly described to be with type-1 or type-2 AIP. In order to better know the diagnosis accuracy associated with serum IgG4 concentration for the diagnosis of IgG4-RD, we reviewed the current evidence to perform this meta-analysis and systematic review.

## METHODS

### Literature Search

We searched MEDLINE, EMBASE, Web of Science, SCOPUS, and Cochrane Library from inception to December 2015. We used the terms “IgG4-related disease” OR “IgG4-related diseases” as text words combined with “serum IgG4” OR “serum immunoglobulin g4.” We also contacted the corresponding authors by e-mail for those unpublished data of some literatures. Studies were excluded if no response was received. All analyses of this systemic review were based on previous published studies, so no ethical approval and patient consent are required.

### Inclusion and Exclusion Criteria

Studies meeting the following criteria were eligible for inclusion: those that assessed the diagnostic accuracy of serum IgG4 for IgG4-RD; those providing detailed diagnostic criteria of IgG4-RD; sufficient data reported to construct a two-by-two table; studies providing the cut-off value for serum IgG4; no language restrictions. The exclusion criteria were: animals, cell cultures, reviews or case reports; duplicate articles; not original articles; <10 IgG4-RD patients or disease controls; insufficient data to construct a two-by-two table; studies focused on AIP which were not clearly described to be type-1 or type-2 were also excluded.

### Data Extraction

The following data were extracted from the included studies: first author; published year; geographical region; disease names and main control diseases; detection method; cut-off value; diagnostic criteria of IgG4-RD; sample size; true positive (TP); false positive (FP); false negative (FN); and true negative (TN). According to the media of sample size, eligible studies were classified as large (≥ median sample size) or small (< median sample size). The accuracy of the data was verified by a second investigator (Liu). Discrepancies between 2 reviewers were resolved by consensus after discussion. The quality of each study was assessed according to the QUADAS-2 (quality assessment of diagnostic accuracy studies 2) by 2 investigators.^18^ Summary of QUADAS-2 plot was generated by the Review Manager Software (version 5.3, the Cochrane Collaboration).

### Statistical Analysis

The pooled sensitivity, specificity, PLR, NLR, DOR, and their confidence intervals (95% CIs) were calculated by the accuracy data (TP, FP, FN, and TN) extracted from each eligible study. The SROC was generated, and the AUC was calculated to represent the overall performance of the detection method.

Spearman rank correlation was used to test for threshold effects, and a *P* value < 0.05 indicated a significant threshold effect. The heterogeneity across included studies caused by the nonthreshold effect was examined by Cochrane Q-statistic and *I*^2^ tests. The *I*^2^ test was used to estimate the extent of heterogeneity. A *I*^2^ value >30% would indicate moderate heterogeneity and a value >50% would represent substantial heterogeneity.^19^ Possible sources of heterogeneity and their effects on the results were explored by subgroup analyses and sensitivity analyses. Prespecified subgroup analyses stratified by sample size, regions, diseases were performed to assess the influence of the above parameters. Deeks’ funnel plot was performed to detect the publication bias. A *P* value <0.10 indicated significant publication bias.

Statistical analysis was performed using the STATA software (version 12.0, Stata Corporation, TX) with the MIDAS module and Meta-DiSc 1.4 (XI, Cochrane Colloquium, Barcelona, Spain). All reported *P* values were 2 sided and *P*<0.05 was considered to be statistically significant.

## RESULTS

### Literature Search

**FIGURE 1:**
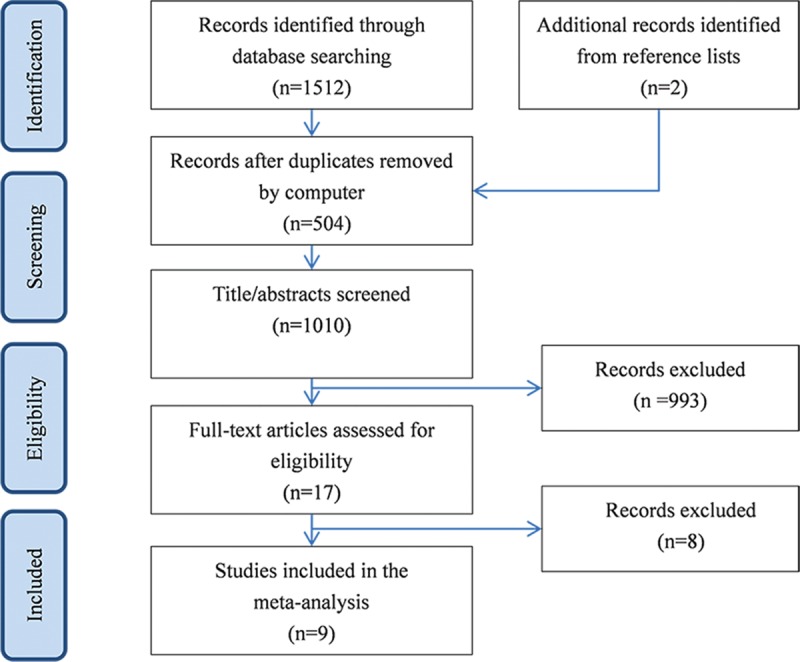
Flowchart of study selection by using the electronic database.

A total of 1514 records were retrieved from initial search on electronic databases, with 321 from EMBASE, 304 from PUBMED, 640 from SCOPUS, 247 from Web of Science, and 2 from the reference lists by hand research. In total, 504 records were removed after duplicates by computer. After title and abstract screening, 993 articles were excluded. Overall, 17 full texts were examined for the eligibility of inclusion. Also, 8 articles were further excluded because they were duplicates, review articles, or insufficient data to construct a two-by-two table. Finally, 9 case-control studies met the inclusion criteria (Figure 1).

### Characteristics of the Included Studies

**FIGURE 2:**
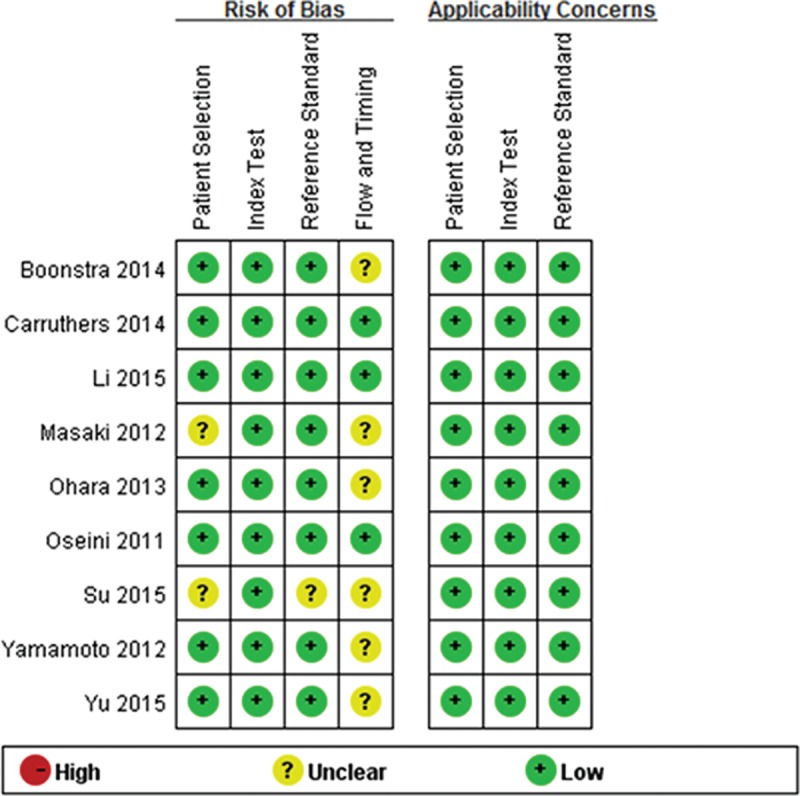
Quality assessment of included studies based on the QUADAS-2. QUADAS-2 = quality assessment of diagnostic accuracy studies 2.

Table 1  presents the main characteristics of the included studies, as well as the diagnostic data of serum IgG4 extracted from each of the studies and analyzed. A total of 9 studies were analyzed, which included 1235 patients with IgG4-RD and 5696 healthy controls. The publication year of the included studies was 2011 or later. With regard to the geographic region of the studies, 2 were carried out in the United States, 1 in Netherland, 3 in Japan, and 3 in China. Figure 2 presents the summary plot of the QUADAS-2. As shown, the methodological quality of the eligible studies was adequate and not significantly affected by bias.

**TABLE 1:**
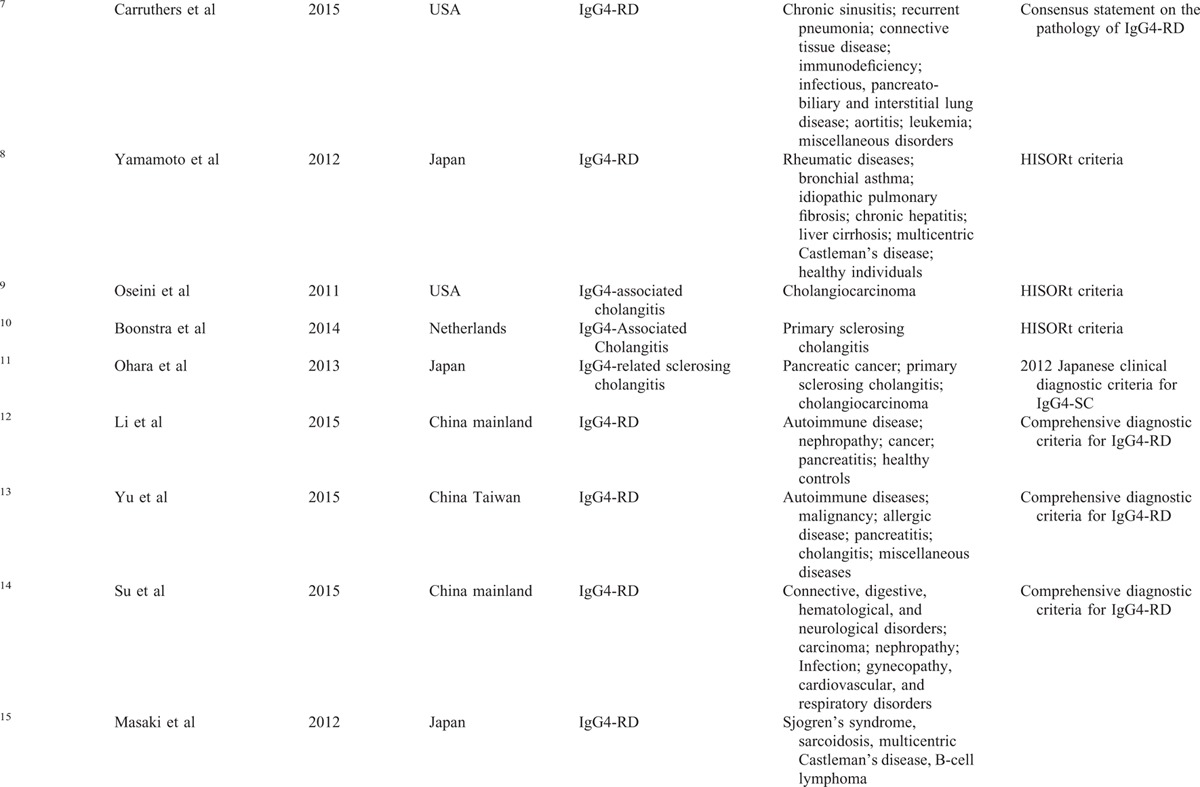
Characteristics and Data Extracted From the Studies Included in the Meta-Analysis

**TABLE 1 (Continued):**
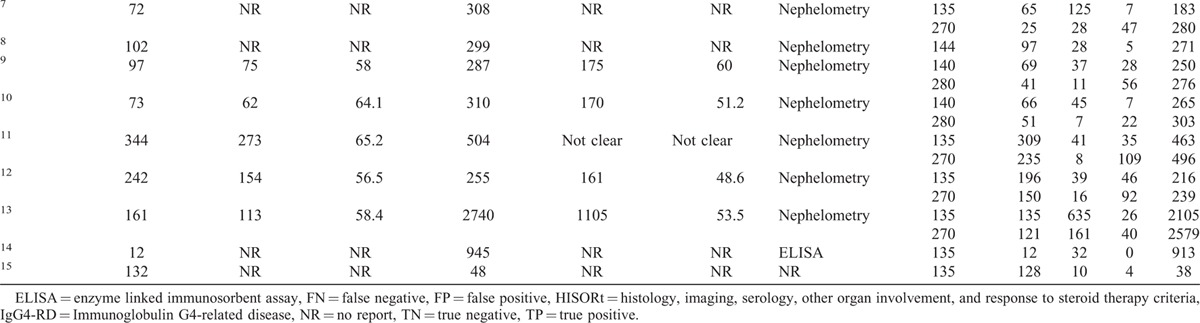
Characteristics and Data Extracted From the Studies Included in the Meta-Analysis

### Diagnostic Accuracy of Serum IgG4

#### Meta-Analysis for a Cut-Off Value of the Upper Limit of Normal

**FIGURE 3:**
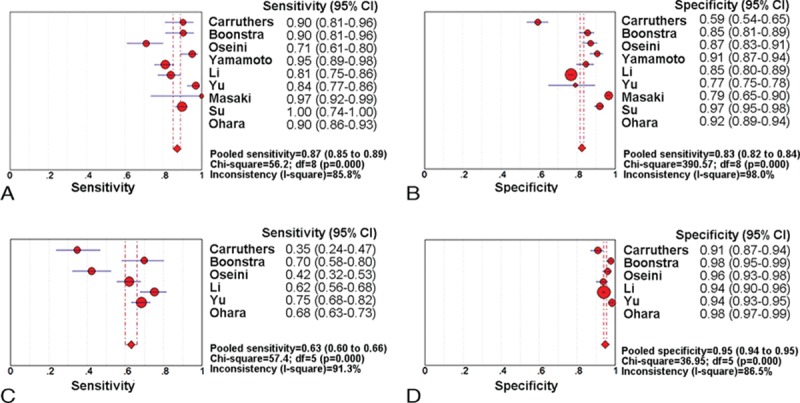
Forest plots of the sensitivity and specificity of serum IgG4 for IgG4-RD diagnosis. Sensitivity (A) and specificity (B) forest plots for a cut-off value of the normal upper limit. Sensitivity (C) and specificity (D) forest plots for a cut-off value of 2-fold the normal upper limit.IgG4-RD = IgG4-related disease.

**FIGURE 4:**
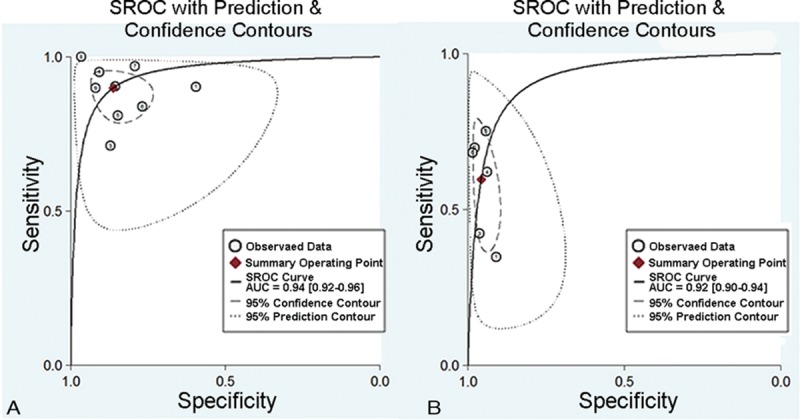
The SROCs of serum IgG4 for IgG4-RD diagnosis. (A) SROC for a cut-off value of the normal upper limit. (B) SROC for a cut-off value of 2-fold the normal upper limit. IgG4-RD = IgG4-related disease, SROC = summary receiver operating characteristic curve.

All the 9 analyzed studies provided a cut-off value of the upper limit of normal between 135 and 144 mg/dL. The reported sensitivity of the serum IgG4 for the diagnosis of IgG4-RD ranged from 71.1% to 100%, and the reported specificity ranged from 59.4% to 96.6%. The pooled estimate, by using a random-effects model, produced an average sensitivity of 87.2% (95% CI, 85.2–89.0%; *Q* = 56.2, *P* < 0.01; *I*^2^ = 85.8%) and an average specificity of 82.6% (95% CI, 81.6–83.6%; *Q* = 390.57, *P* < 0.01; *I*^2^ = 98%). Additionally, the average PLR and NLR were 6.48 (95% CI, 3.98–10.57; *Q* = 311.3, *P* < 0.01; *I*^2^ = 97.4%) and 0.14 (95% CI, 0.09–0.21; *Q* = 50.77, *P* < 0.01; *I*^2^ = 84.2%), respectively. The DOR was 45.15 (95% CI, 23.41–87.06; *Q* = 64.53, *P* < 0.01; *I*^2^ = 87.6%). The AUC of the SROC was 0.94 (0.92–0.96). The sensitivity and specificity forest plots and SROC curve were shown in Figure 3A and B, and Figure 4A, respectively.

#### Meta-Analysis for a Cut-Off Value of 2-Fold the Upper Limit of Normal

In total, 6 of 9 studies in our meta-analysis provided the diagnostic values of 2-fold the upper limit of normal between 270 and 280 mg/dL. These studies included a total of 989 patients with IgG4-RD and 4404 overall controls. The reported sensitivity of the serum IgG4 for diagnosis of IgG4-RD ranged from 34.7% to 75.2%, and the reported specificity ranged from 90.9% to 98.4%. The pooled estimate, by using a random-effects model, produced an average sensitivity of 63% (95% CI, 60.0–66.0%; *Q* = 57.4, *P* < 0.01; *I*^2^ = 91.3%) and an average specificity of 94.8% (95% CI, 94.1–95.4%; *Q* = 36.9, *P* < 0.001; *I*^2^ = 86.5%). Additionally, the average PLR and NLR were 13.3 (95% CI, 7.39–24.0; *Q* = 48.51, *P* < 0.001; *I*^2^ = 89.7%) and 0.41 (95% CI, 0.29–0.58; *Q* = 90.16, *P* < 0.001; *I*^2^ = 94.5%), respectively. The DOR was 33.42 (95% CI, 13.88–80.43; *Q* = 60.51, *P* < 0.001; *I*^2^ = 91.7%) and AUC of the SROC was 0.92 (0.90–0.94). The forest plots and SROC were shown in Figure 3C and D, and Figure 4B, respectively.

### Threshold Effect and Heterogeneity

No significant threshold effect was found by visual assessment of SROC curve (Figure 4A and B). The Spearman correlation coefficient was further performed to confirm the effect. The Spearman correlation coefficient was –0.167 (*P* = 0.668) and –0.486 (*P* = 0.329) for the 2 cut-offs of serum IgG4, respectively, which suggested the absence of threshold effect. Considering the significant heterogeneity among studies, we performed subgroup analyses to explore the source of heterogeneity. Sample size, regions, and diseases were analyzed for each accuracy data. However, none of these above was identified as a possible source of heterogeneity. Nevertheless, the diagnostic accuracy data were consistent across different subgroups (Table 2).

**TABLE 2:**
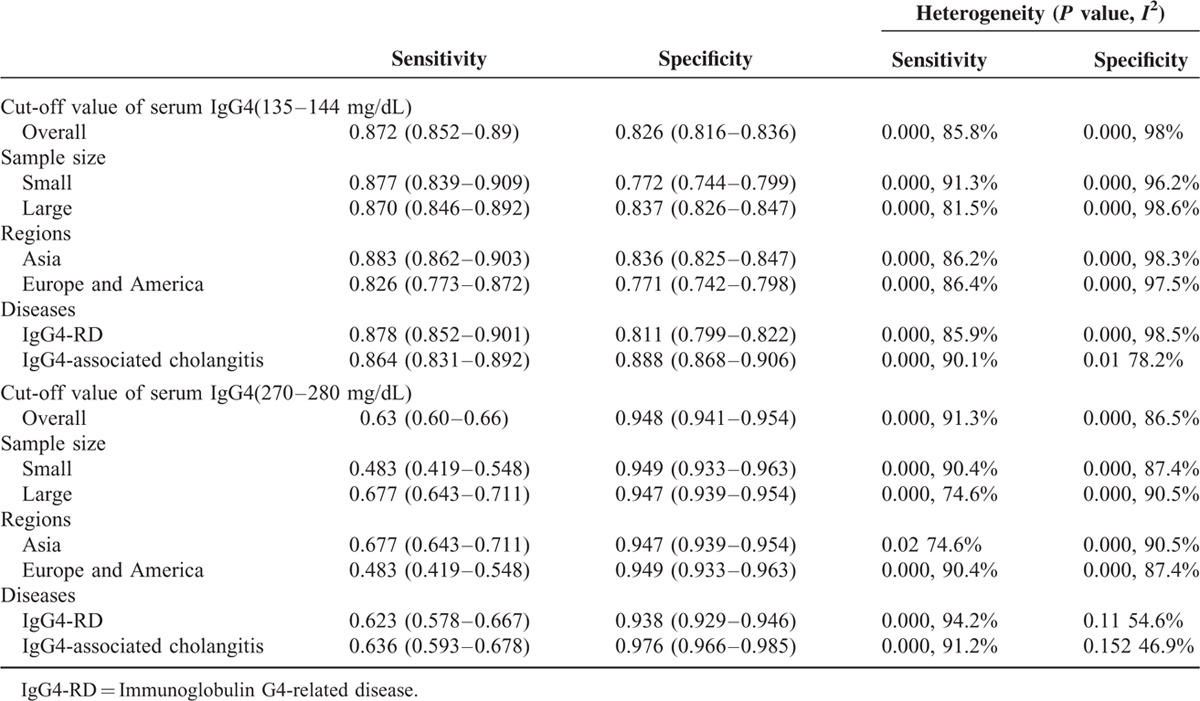
Subgroup Analysis of the 9 Included Studies

### Sensitivity Analysis and Publication Bias

**FIGURE 5:**
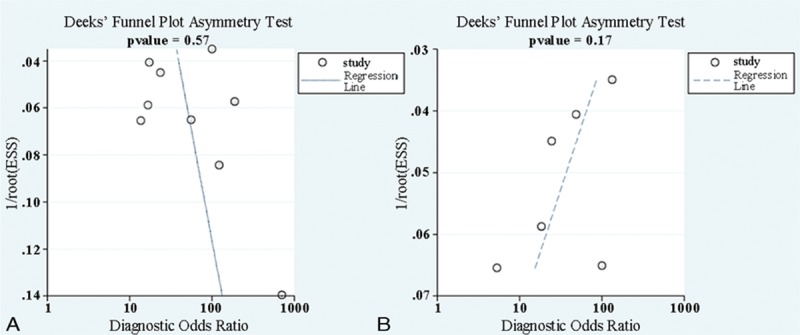
Publication bias of serum IgG4 for IgG4-RD diagnosis. Funnel plot for the cut-off value of the normal upper limit (A) and 2-fold the normal upper limit (B). IgG4-RD = IgG4-related disease.

Significant heterogeneity could be detected from the results of diagnostic accuracy tests. We did sensitivity analysis by omitting a single study, but the results showed the pooled results were not significantly affected by individual studies, indicating a good stability of the meta-analysis (Table 3). As shown in Figure 5A and B, Deeks’ funnel plots were almost symmetric and *P* values were 0.57 and 0.17 for the 2 cut-off values of serum IgG4, respectively, which suggested a low likelihood of publication bias.

**TABLE 3:**
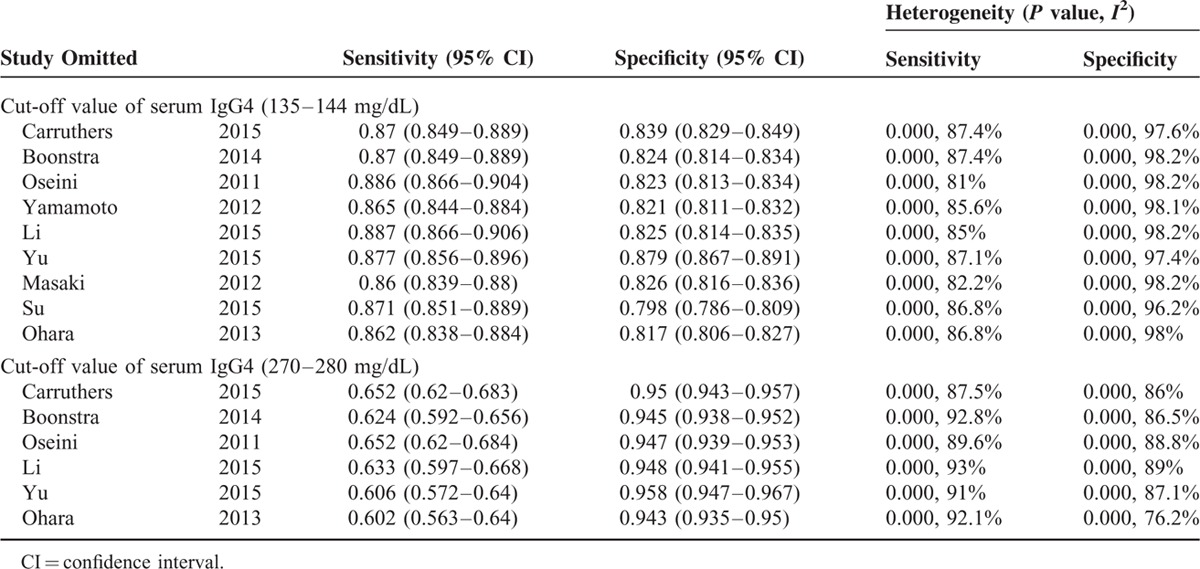
Meta-Analysis Estimates, Name Given to Study is Omitted

## DISCUSSION

A serum IgG4 concentration >135 mg/dL has been widely accepted as the cut-off value for the diagnosis of IgG4-RD. In this analysis, all the 9 studies provided the diagnostic values of the upper limit of normal. By pooling the data from these studies that included a total of 6931 subjects, we calculated the pooled sensitivity for serum IgG4 in the diagnosis of IgG4-RD was 0.872 and the specificity was 0.826. The DOR is a single indicator of test performance derived from sensitivity and specificity, which is defined as the ratio of the odds of positivity in disease relative to the odds of positivity in the nondiseased.^20^ Higher values indicate better discriminatory test performance. Our analysis showed the pooled DOR value was 45.15, which means that the serum IgG4 is a useful biomarker in diagnosing IgG4-RD. In addition, the AUC of SROC with a value of 0.94 also showed good accuracy of serum IgG4.

Considering that this cut-off level displayed lower specificity in discriminating IgG4-RD, we also performed a meta-analysis of serum IgG4 at a cut-off value of >2 times the upper limit of normal. Among 9 studies included in our meta-analysis, 6 provided the diagnostic accuracy. The pooled specificity of serum IgG4 was higher (94.8%), but the sensitivity was lower (63%). The pooled DOR was 33.42 and the AUC of SROC was 92.17, which were not improved in comparison with the conventional cut-off value. The higher specificity derived from the higher cut-off value for serum IgG4 concentration, however, came at a significant reduction in sensitivity.

In addition, 2 of the enrolled studies investigated the diagnostic value of serum IgG4 with total serum IgG (IgG4/IgG) ratios for IgG4-RD. One found that the ROC curve of serum IgG4/IgG ratio was almost identical to the serum IgG4 concentration.^15^ The other study by Carruthers et al^7^ showed neither the specificity nor the positive predictive value was improved by the serum IgG4/IgG ratio for the diagnosis of IgG4-RD. These finding supported that the IgG4/IgG ratio could not improve the overall test characteristics compared with serum IgG4 alone. To better elucidate the usefulness of serum IgG4/IgG ratio, further studies are needed.

Substantial heterogeneity in the pooled accuracy data was found among studies. Subgroup analysis was performed according to sample size, regions, and diseases. But unfortunately, none of the analyzed covariates was the source of heterogeneity. We also performed sensitivity analyses and found that the pooled results were relatively stable and not affected by a single study. As we know, IgG4-RD is a systematical disorder involving numerous organs including the pancreas, salivary glands, retroperitoneum, kidneys, lymph nodes, and others. Additionally, different case controls were enrolled according to the purposes and the interested diseases. The heterogeneity could possibly be caused by difference in selection of IgG4-RD cases and controls.

Our findings were strengthened by excluding the studies concerning the diagnostic value of serum IgG4 for AIP, in which the subtypes of AIP patients were not clearly described. AIP could be classified into 2 types according to the international consensus diagnostic criteria (ICDC).^21^ Only type 1 AIP is part of the spectrum of IgG4-RD. In a previous meta-analysis, Morselli-Labate and Pezzilli^16^ reported the pooled sensitivity and specificity of serum IgG4 in diagnosing AIP were 82.4% and 94.6%, respectively.^16^ Similar findings were reported in a recent study by Lian et al.^17^ The results indicated that serum IgG4 exhibited high specificity (94%) and relatively low sensitivity (74%). However, the subtypes of these AIP patients in the included studies were not clearly described, which might explain the difference to our findings. Furthermore, although no publishing years were set during our literature searching, only publications after 2011 were included in this meta-analysis. This was consistent with that the newly emerged disease was referred to as IgG4-RD only more than a decade ago.^22^ More detailed diagnostic criteria were proposed which can ensure the selection of patients with IgG4-RD and overall controls.

Several limitations in this meta-analysis need to be acknowledged. First, only a relatively small number of studies with limited subjects were included in the meta-analysis, which may reduce the statistical power for determining the diagnostic role of serum IgG4. Second, as mentioned above, significant heterogeneity was observed in this analysis. Because the number of included studies was <10, a multivariate meta-regression was not performed. New studies should be specifically designed in order to identify the possible confounding factors.

In conclusion, this study is the first to assess the diagnostic accuracy of serum IgG4 in patients with IgG4-RD using meta-analysis, which provided evidence that serum IgG4 is a modestly effective marker for the disease. Doubling the cut-off value for IgG4 could improve the specificity, but inevitably accompany with a significant sacrifice in sensitivity.
